# Gold Nanoparticle-Based Colorimetric Strategies for Chemical and Biological Sensing Applications

**DOI:** 10.3390/nano9060861

**Published:** 2019-06-06

**Authors:** Chia-Chen Chang, Chie-Pein Chen, Tzu-Heng Wu, Ching-Hsu Yang, Chii-Wann Lin, Chen-Yu Chen

**Affiliations:** 1 Biomedical Technology and Device Research Laboratories, Industrial Technology Research Institute, Hsinchu 310, Taiwan; cwlinx@ntu.edu.tw; 2 Department of Obstetrics and Gynecology, Mackay Memorial Hospital, Taipei 104, Taiwan; cpchen@mmh.org.tw; 3 Graduate Institute of Biomedical Electronics and Bioinformatics, National Taiwan University, Taipei 106, Taiwan; aresation@gmail.com (T.-H.W.); d02945009@ntu.edu.tw (C.-H.Y.); 4 Department of Biomedical Engineering, National Taiwan University, Taipei 106, Taiwan

**Keywords:** gold nanoparticle, colorimetric assay, aggregation of AuNPs, etching of AuNPs, growth of AuNPs, nanoenzyme

## Abstract

Gold nanoparticles are popularly used in biological and chemical sensors and their applications owing to their fascinating chemical, optical, and catalytic properties. Particularly, the use of gold nanoparticles is widespread in colorimetric assays because of their simple, cost-effective fabrication, and ease of use. More importantly, the gold nanoparticle sensor response is a visual change in color, which allows easy interpretation of results. Therefore, many studies of gold nanoparticle-based colorimetric methods have been reported, and some review articles published over the past years. Most reviews focus exclusively on a single gold nanoparticle-based colorimetric technique for one analyte of interest. In this review, we focus on the current developments in different colorimetric assay designs for the sensing of various chemical and biological samples. We summarize and classify the sensing strategies and mechanism analyses of gold nanoparticle-based detection. Additionally, typical examples of recently developed gold nanoparticle-based colorimetric methods and their applications in the detection of various analytes are presented and discussed comprehensively.

## 1. Introduction

Simple and convenient technologies for the identification of chemical and biological species are of great significance in environmental monitoring, public health, and disease diagnosis [1,2]. However, detecting these chemical and biological species rapidly and cost-effectively with high sensitivity and specificity is challenging. Traditional sensing techniques, such as chromatography, electrochemistry, field-effect transistors, surface plasmonic resonance sensors, and microgravimetric methods often require relatively expensive, cumbersome instruments and a skilled professional to operate them in order to detect the various targets. For home testing or on-site analysis, there is still scope for improvement in these techniques [3,4,5,6]. Compared with the various traditional detection methods, colorimetric assays are extremely attractive due to their convenience, simplicity, and cost-effectiveness [7]. Moreover, the colorimetric response is easy to monitor with the naked eye without any sophisticated instrumentation; this assay is thus suitable for on-site detection [8]. 

Gold nanoparticle (AuNP)-based colorimetric sensors are of particular interest due to the several unique features of gold nanomaterials, such as complex optical properties, controllable size, and catalytic properties [9], and the synthesis of AuNPs is efficient and straightforward [10]. Most importantly, the versatile surface chemistry of AuNPs provides a significant linkage capability toward a wide range of molecular probes with thiol groups for the functional conjugation and detection of chemical and biological targets [11,12,13,14]. Each of these AuNP features has motivated significant efforts for the development of a new generation of sensing strategies with enhanced sensitivity, specificity, and stability. In the last two decades, AuNP developments have generated a dramatic increase in innovative colorimetric approaches for the efficient detection of nucleic acids [15,16,17], proteins [18,19,20], and small molecules [21,22,23].

**Figure 1:**
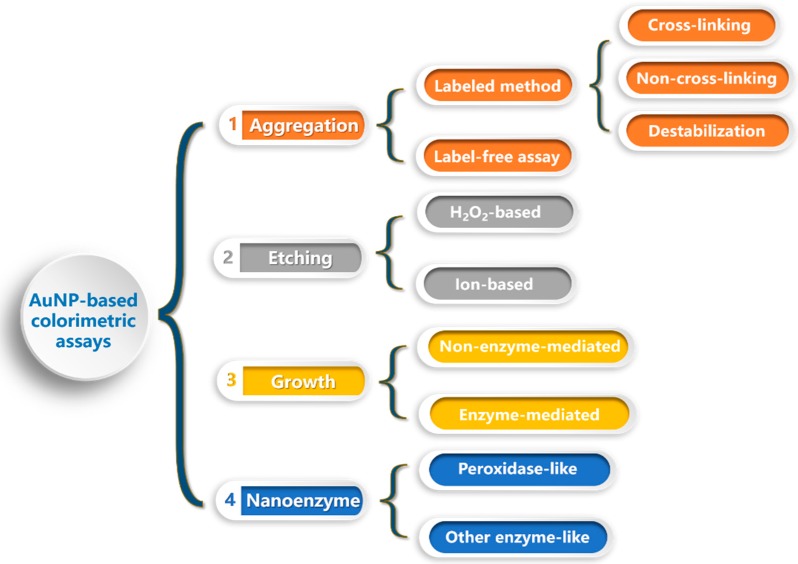
The summary of different types of gold nanomaterial-based colorimetric sensors.

We believe that a short review of the recent developments (2014–2018) in AuNP-based colorimetric assays is necessary to facilitate further research into this topic, but hundreds of relevant papers from the past five years are left out. The purpose of this review is to briefly introduce the new progress in areas ranging from novel sensing concepts and signal amplification strategies for representative sensing applications. The discussion is organized by the type of AuNP-based colorimetric sensors (summarized in Figure 1), starting with aggregation-based sensors followed by etching-, growth-, and nanozyme-based sensors. Finally, future challenges and perspectives for the development of gold nanomaterial-based colorimetric analyses will be discussed. 

## 2. Aggregation-Based Colorimetric Assays

Because AuNPs possess unique localized surface plasmon resonance (LSPR) properties with high molar extinction coefficients, they show size-dependent, distinct color changes. Generally, a colloidal solution of 20 nm AuNPs has a wine-red color, and the LSPR band of the 20 nm AuNPs occurs at approximately 520 nm. The aggregation of AuNPs results in a large red-shift of the LSPR peak, accompanied by a characteristic color change from red to blue [24]. Therefore, aggregation-based colorimetric assays have wide-ranging sensing applications in proteins [25], small molecules [26], inorganic ions [27,28], enzyme activities [29], and oligonucleotides [30]. Currently, these colorimetric assays can be divided into two systems: labeled and label-free.

### 2.1. Labeled Detection Methods 

The labeled method directly attaches ligands such as DNA, aptamers, peptides, or antibodies onto AuNPs through chemical linkages prior to detection. Ligand-modified AuNPs have higher steric and hydration-based interparticle repulsions and are more stable at high ionic strength conditions than bare AuNPs, which are unstable and undergo aggregation due to the salt-induced screening effect. However, the controlled aggregation of AuNPs in label-based colorimetric strategies can be achieved by cross-linking, non-cross-linking, or destabilization aggregation.

#### 2.1.1. Cross-Linking Aggregation

**Figure 2:**
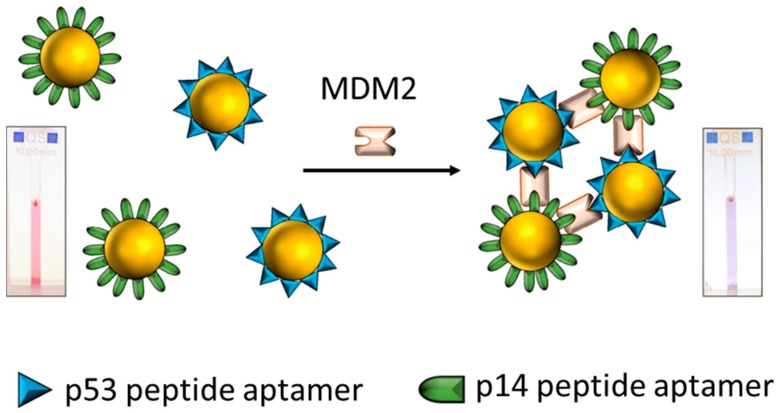
The schematic representation of the detection of Mdm2 using two peptide aptamer- functionalized AuNPs. After the addition of Mdm2, the aggregation of the AuNPs was driven by the formation of a ternary complex of Mdm2, p53, and p14, and the color of the solution changed from red to blue. Reproduced with permission from [37]. American Chemical Society, 2017.

In cross-linking strategies, AuNP aggregation is induced by the controlled assembly of ligand-functionalized AuNPs with the formation of intermolecular bonds (such as H-bonding or hydrophobic interactions), which overpower the interparticle repulsive forces (such as electrostatic repulsion). Peptide-functionalized AuNPs have been developed as biological recognition probes for cross-linking methods in colorimetric diagnoses [31,32,33,34,35]. Chandrawati et al. utilized peptide-conjugated AuNPs for monitoring the concentration of blood coagulation Factor XIII, which requires thrombin and Ca^2+^ for activation [36]. The peptides were terminated in glutamine or lysine residues, allowing them to be linked by the formation of an amide bond in the presence of active Factor XIII. Thus, this cross-linking reaction causes a notable decrease in interparticle distances, which results in the aggregation of AuNPs. Using a similar approach, Retout et al. developed a one-step method for the rapid detection of oncoprotein Mdm2 (Figure 2), which is a p53- and p14-binding protein and which may act as a regulator of these protein functions [37]. Hence, the AuNPs were modified with the peptides of proteins p53 and p14. After the addition of Mdm2, the aggregation of the AuNPs was driven by the formation of a ternary complex of Mdm2, p53, and p14, and the color of the solution changed from red to blue. In addition to protein detection, Yang et al. reported the monitoring of Hg^2+^ and As^3+^ by the modification of peptide A3 (AYYSGAPPMPPF) to AuNPs [38]. The metal ions induced the assembly of the AuNPs as the interparticle distances changed due to the complexation of the metal ions with the peptide A3. Elsewhere, Ding et al. used peptide-immobilized AuNPs for the detection of trypsin [39]. Substrate peptides (YHPQMNPYTKAGGGC) triggered the cross-linking aggregation of AuNPs through their cysteine and lysine residues and the hydrolysis of the substrates by trypsin inhibited the aggregation of AuNPs. Using this method, they reported a detection limit of 0.5 nM for trypsin. 

**Figure 3:**
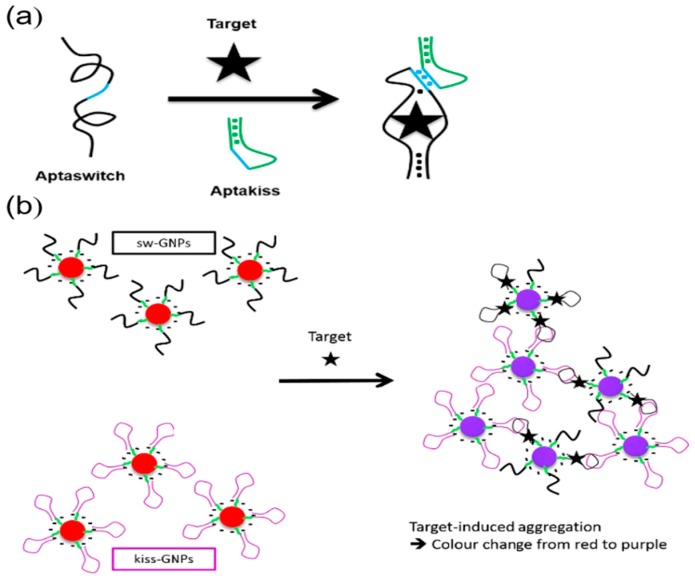
(**a**) The schematic illustration of the formation of an aptamer kissing complex. The loop-loop interaction is shown in blue. (**b**) The principle of the cross-linking aggregation of AuNPs induced by the aptamer kissing complex. Reproduced with permission from Reference [47]. Royal Society of Chemistry, 2017.

Other ligands such as nucleic acids [40,41], aptamers [42,43], and antibodies [44,45] have been widely used to stabilize and functionalize AuNPs for the development of colorimetric immunosensors. For example, Lesniewski et al. modified the AuNPs using antibodies for the one-step detection of the virus T7 bacteriophage [46]. This method showed a fast response time and a comparable sensitivity compared to the standard plaque test. Goux et al. introduced a new cross-linking strategy (Figure 3) that relied on the formation of kissing complexes for sensing adenosine molecules [47]. Kissing complexes occur in RNA–RNA interactions which allow for the assembly of complementary loop domains through Watson–Crick base pairing [48]. In this study, they designed a target-responsive kissing aptamer, called aptaswitch, which has an adenosine-binding region and a kissing region for binding to another RNA loop (aptakiss). In the presence of the target, adenosine specifically bound to its aptamer and changed the aptamer conformation from a random-coil structure to a hairpin-like structure, thereby generating the aptaswitch. Then, the aptakiss could establish a specific loop-loop interaction with the aptaswitch, resulting in the cross-liking aggregation of AsNPs and a red-to-purple color change. Due to the variety of hairpin aptamer probes and kissing motifs, this strategy opens an opportunity for developing further colorimetric sensing platforms.

Guo et al. [49] introduced the concept of asymmetrical DNA–AuNP oriented aggregation. Unlike conventional cross-linking aggregation methods that form large nanoparticle aggregates, Guo et al. formed AuNP dimers, which effectively improved the long-term stability of AuNPs. Due to a remarkable decrease in the interparticle gap caused by the AuNP dimers, this method provided a 100-fold wider dynamic range of detection and a 10,000-fold lower limit of detection (LOD) as compared with conventional colorimetric sensors. In addition to DNA detection, this oriented aggregation has been used for the analysis of melamine [50], aspartic acid [51], microcystin-LR [52] and nitrite ions [53].

Cross-linking aggregation methods offer a convenient colorimetric assay platform. Nevertheless, the sensitivity is limited to the nanomolar or subnanomolar levels due to the lack of amplification. In most cases, the concentrations of molecular biomarkers are found in picomolar levels in healthy individuals; therefore, these markers are undetectable by cross-linking aggregation. To improve the sensing performance, various signal amplification methods have been rationally designed and combined with the cross-linking aggregation, including enzyme-aided signal amplification (e.g., exonuclease [54,55], nicking endonucleases [56,57], and polymerases [41,58]) and enzyme-free amplification (e.g., hybridization chain reaction [59,60] and catalytic hairpin assembly [61,62]) strategies. With the use of these amplification approaches, the sensitivity has reached the required levels of sensitivity and enabled picomolar and even attomolar detection capabilities for satisfactory diagnostic tests.

#### 2.1.2. Non-Cross-Linking Aggregation

**Figure 4:**
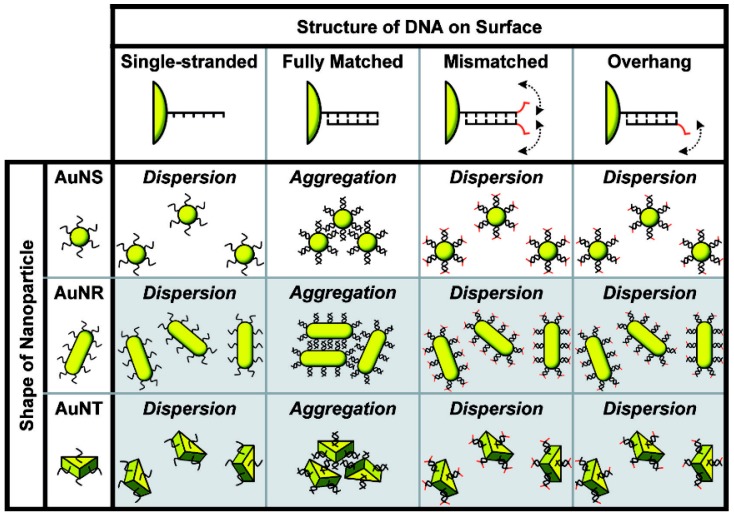
The different colloidal behaviors of ssDNA-labeled gold nanospheres, nanorods (AuNRs), and nanotriangles in a moderately high ionic strength medium. Double-headed arrows indicate unpaired mononucleotides. Reproduced with permission from Reference [68]. Wiley, 2015.

Double-stranded DNA (ds)-meditated AuNP assembly at high ionic strength was demonstrated by Maeda’s group [63]. When the AuNPs are modified with single-stranded DNA (ssDNA), the dispersion of the ssDNA–AuNP complex in an aqueous medium of high ionic strength is stable due to interparticle electrostatic and steric repulsion between the ssDNA-modified AuNPs (ssDNA-AuNPs). The formation of dsDNA–AuNP complexes results in a spontaneous aggregation of the AuNPs within several minutes, and a red-to-purple color change. This transition reduces the electrostatic interaction between nanoparticles because the rigid structures of the duplexes favor binding with counter cations. Moreover, duplexes lower the entropic effect, which decreases the steric repulsion [64,65]. Notably, the dsDNA–AuNPs with a single-pair mismatch at the terminal position significantly increase the colloidal stability of the AuNPs and prevent the non-cross-linking aggregation. Based on this finding, the unique dispersion behavior of dsDNA–AuNPs has been successfully applied in the rapid colorimetric detection of various targets [66,67]. In addition, these colloidal behaviors are found in different sizes (5–300 nm) and shapes (sphere, rod, and triangle; Figure 4) of nanoparticles originating from the multiple blunt-end stacking interaction to dominate the interparticle attractive forces [68,69]. 

Currently, DNA hybridization using ssDNA–AuNPs has been explored by both cross-linking and non-cross-linking methods and the rapidity of the solution color change of the two colorimetric strategies were compared. In a large number of target DNAs, the non-cross-linking aggregation occurred significantly faster than with the cross-linking aggregation. In contrast, when a small number of DNA was provided, the non-cross-linking aggregation showed a much slower color change than the cross-linking aggregation [70]. This study by Maeda’s group will serve as a guide to help future researchers choose the appropriate aggregation strategies for designing DNA–AuNP-based colorimetric sensors under given conditions.

**Figure 5:**
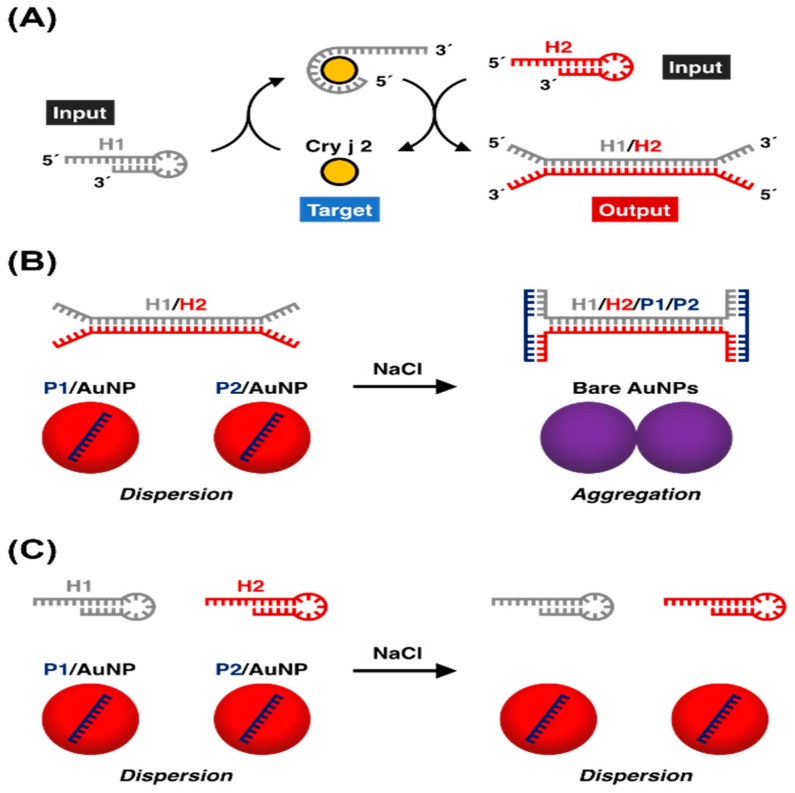
A non-crosslink aggregation assay showing the principle of catalytic target recycling. (**a**) The Cry j 2 target catalyzes the reaction of the hairpins H1 and H2 into a duplex structure, to produce an H1/H2 duplex after each cycle. (**b**) In the presence of Cry j 2, the H1/H2 duplex forms a three-way DNA junction with P1. Thus, the color does not change due to the colloidal stabilization of ssDNA–AuNPs. (**c**) In contrast, the red-to-purple color change is caused by salt-induced non-cross-linking aggregation of dsDNA−AuNPs in the absence of Cry j 2. Reproduced with permission from Reference [71]. American Chemical Society, 2019.

Likewise, non-cross-linking aggregation methods also suffer from sensitivity issues. To overcome this limitation, Maeda’s group developed a plasmonic colloidal nanosensor through the combination of signal amplification using catalytic DNA hairpin self-assembly and signal transduction using the salt-induced aggregation of DNA-modified AuNPs (Figure 5) [71]. The detection limit was 130-fold greater than that of previously reported methods. In this study, they employed an anti-Cry j 2 DNA aptamer as a molecular recognition unit, which accounted for the lack of false responses to non-target allergen proteins. In addition, the assay was robust enough to enable the detection of Cry j 2 spiked soil solutions without any interference from the contaminants. This method could be readily applied to the visual detection of various proteins by using appropriate aptamers as recognition units.

#### 2.1.3. Destabilization-Induced Aggregation

**Figure 6:**
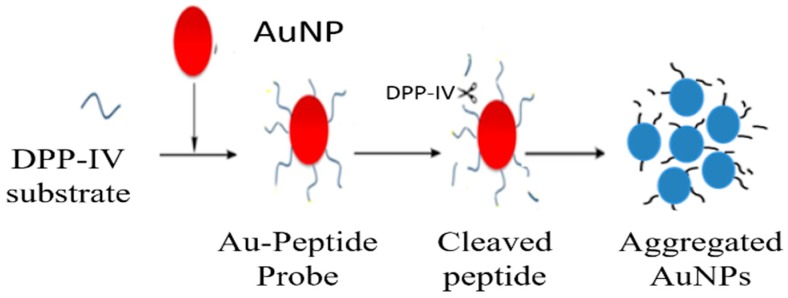
The schematic representation of the measurement of dipeptidyl peptidase-IV(DPP-IV) activity. DPP-IV hydrolyzes its substrate, which abolishes electrosteric stabilization, which destabilizes the AuNP sensing system and causes AuNPs aggregation. Reproduced with permission from Reference [73]. MDPI, 2018.

Apart from the non-cross-linking aggregation strategies described above, the change of ligand length or structure on the AuNP surface can also be used to control the AuNP aggregation. In the destabilization-induced aggregation strategies, AuNP aggregation is induced by the controlled loss of electrosteric stabilizations when a part of the ligand is cleaved. For example, McVey et al. reported a method to detect pathogenic bacterial DNA by using RNase H-controlled aggregation of AuNPs. RNA-functionalized AuNPs (RNA–AuNPs) were cleaved by RNase H when the formation of DNA–RNA hybridization [72]. Because RNase H catalyzed the cleavage of RNA in a DNA–RNA complex, the DNA target could be liberated and hybridize with other RNA probes on the AuNPs. Although this strategy using RNA–AuNPs is simple and provides high sensitivity, the experimental conditions should be carefully chosen because RNA is a labile material that degrades extremely rapidly, which makes it difficult material with which to work. Similarly, Aldewachi optimized a colorimetric assay for the real-time monitoring of dipeptidyl peptidase-IV activity based on the hydrolysis of peptide functionalized AuNPs in the presence of an enzyme (Figure 6) [73,74]. Zhang then utilized this strategy for the real-time measurement of lipase kinetics, using Tween 20 as a stabilizing substrate for lipase. Firstly, the addition of Tween 20 can adsorb onto the surface of AuNPs to prevent the aggregation of AuNPs. The lipase can catalyze the hydrolysis of Tween 20 and cause the formation of unstable Tween 20-modified AuNPs. Thus, the resultant aggregation and red-to-blue color change exhibited the presence of lipase [75].

### 2.2. Label-Free Detection Methods 

**Figure 7:**
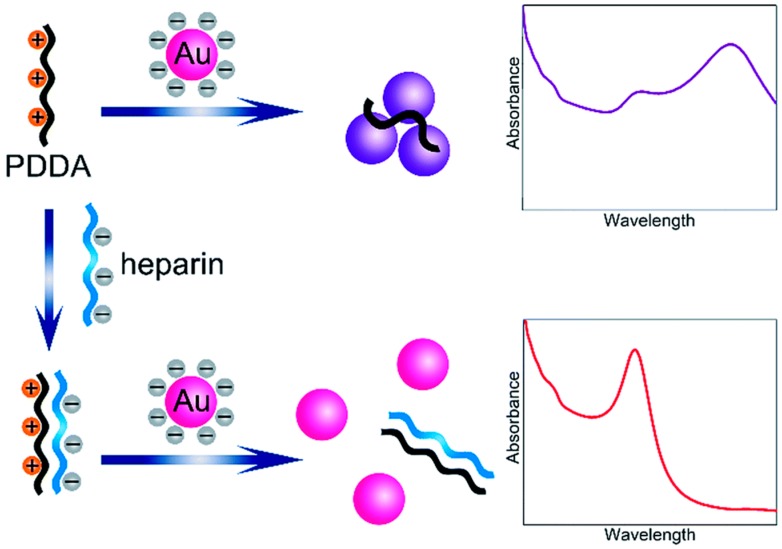
The schematic illustration of heparin detection using a label-free colorimetric method. After the addition of poly(diallyldimethylammonium chloride) (PDDAs), AuNPs aggregate due to attractive electrostatic interactions between the PDDA and the AuNPs. However, when heparin is present, the PDDA molecules will interact with the heparin, which prevents the PDDA-induced aggregation of the AuNPs. Reproduced with permission from Reference [76]. Royal Society of Chemistry, 2019.

The aggregation of functionalized AuNPs can be triggered by the loss of steric or electrosteric stabilization or by the gain of the stacking interaction. Unlike the label-based detection methods, the label-free colorimetric methods are mostly regulated by electrostatic stabilization. In the case of electrostatic stabilization, a repulsive electric layer can be generated from the surface charges of AuNPs to stabilize colloids. Thus, the neutralization of surface charges, in this case, results in the formation of unstable AuNPs, which promotes aggregation and a red-to-purple color change. The analyte-triggered aggregation for chemical and biological sensing applications is performed based on the affinity of analytes such as the electrostatic or hydrogen-bonding interaction toward unmodified AuNPs. For example, Ma et al. devised a simple assay for the sensitive and selective colorimetric detection of heparin [76]. In the absence of heparin, the poly(diallyldimethylammonium chloride) (PDDA) with positive charges can easily adsorb onto the citrate-capped AuNP surfaces by electrostatic attraction (Figure 7). This behavior leads to the aggregation of the AuNPs and a corresponding red-to-blue color change. On the other hand, heparin bears a high negative charge density, which can strongly bind PDDA via an electrostatic interaction to form a stable complex. Therefore, the PDDA induced aggregation of AuNPs could be effectively inhibited by heparin. 

Among various label-free colorimetric detection methods, the combination of aptamers with unmodified AuNPs have been popular for many analytes due to the ease of detection. Generally, the negatively charged aptamers are adsorbed onto the surface of AuNPs via the DNA nitrogen bases, which leads to well-dispersed, negatively charged, aptamer-capped AuNPs in the media of moderately high ionic strength. However, the target triggered configuration swift within the aptamer results in the detachment of the aptamer from the AuNP surface by decreasing the aptamer affinity to AuNPs. As a result, the degree of AuNP aggregation is a direct reflection of the target concentration and can be observed by the red to purple-blue color change in the AuNP solution. Based on the similar strategies, the label-free colorimetric detection of various targets, including proteins [77,78,79,80], small organic molecules [81,82,83], and ions, such as silver (I) [84], cadmium (II) [85] and potassium (I) [86], has been explored extensively.

**Figure 8:**
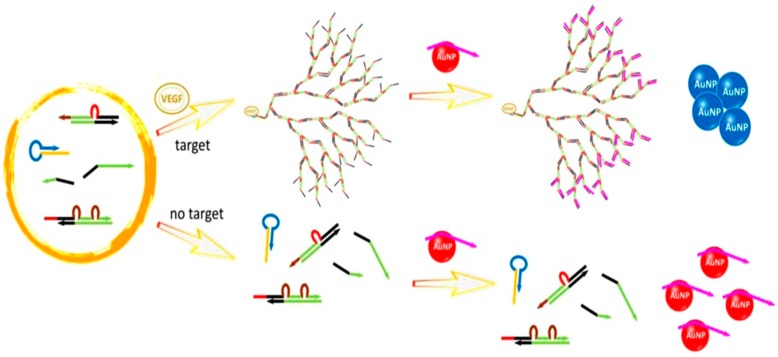
The schematic illustration of target-induced branched DNA cascade reaction and sensing using unmodified AuNPs. Reproduced with permission from Reference [87]. Elsevier B.V., 2016.

These colorimetric approaches are simple and do not require the surface modification of the AuNPs, but their sensitivity is still unsatisfactory. To overcome this drawback, we developed an amplified aptamer detection method by the combination of catalytic hairpin assembly (CHA) and unmodified AuNPs [62]. A dominant merit of CHA over other amplification approaches, such as the polymerase chain reaction (PCR), is that it allows for specific non-enzymatic self-assembly at room temperature. Unlike PCR, the current form of CHA also offers linear reaction kinetics. We recently reported a nonlinear DNA self-assembly system that provides a higher amplification efficiency and more rapid amplification kinetics [87]. A programmed dendritic DNA nanostructure was generated using two double-stranded substrates and two single-stranded helpers as DNA assembly components by a target-induced cascade reaction. Then, this nanostructure was captured by DNA probe-capping AuNPs. The release of the DNA probes from AuNPs led to the aggregation of AuNPs (Figure 8). By using this method, a concentration as low as 3.7 fmol of vascular endothelial growth factor (VEGF), which was much lower than that of other aptamer sensors, could be detected within an hour.

## 3. Etching-Based Colorimetric Detection

Presently, various AuNPs, such as gold nanospheres, gold nanorods (AuNRs) [88,89], gold nanotriangles, and gold nanourchins [88] have been used as etching-based colorimetric sensors. Generally, the etching process causes a change in the shape or size of the nanoparticles and therewith a shift in the LSPR extinction peak and a subsequent change in color. For example, AuNRs have two separate SPR absorption peaks defined as a transverse and a longitudinal mode, respectively [90]. The longitudinal surface plasmon of AuNRs is strongly dependent on the aspect ratio of the nanorods. As the aspect ratio decreases, the longitudinal peak of the AuNRs shows a characteristic blue shift. Therefore, different optical properties can be obtained by properly tuning the aspect ratio to change the wavelength range of the optical properties of the AuNRs. Based on these shape-induced anisotropic optical properties, AuNRs are more ideal gold nanostructures for etching-based sensing strategies, and because their tips have a high surface energy, the etching reaction occurs easily at the AuNR tips. The use of etchants like H_2_O_2_ and I^−^ ions can preferentially etch the terminal ends of AuNRs, which leads to a lower aspect ratio or spherical AuNPs. Moreover, an obvious blue shift in the longitudinal mode of the SPR peak and color change from green to red will be observed. 

### 3.1. H_2_O_2_-Mediated Etching Reactions

Although the etching of AuNRs by H_2_O_2_ has been reported [91], there are several drawbacks in the reaction conditions, such as the high H_2_O_2_ concentrations, high temperature, and acidic environments, thereby limiting the applicability of this system for sensing purposes. However, the introduction of catalytic species such as metal ions and enzymes could overcome this issue [92]. Zhang et al. [93] developed a Co^2+^ sensor based on Fenton-like reaction-mediated etching of AuNRs. In this approach, Co^2+^ induced the decomposition of H_2_O_2_ to generate hydroxyl radicals and these radicals etched the AuNRs in the presence of SCN^−^ and the AuNRs underwent a shape conversion from rods to spheres with an obvious color change from green to red. Under optimized conditions, this assay could visually detect Co^2+^ at 40 nM within 7 min. Due to its good sensing performance in terms of sensitivity, selectivity, and detection time, this method has the potential for the on-site monitoring of Co^2+^. In another recent study conducted by Weng et al. [94], cetyltrimethylammonium bromide (CTAB) was found to accelerate the rate of the H_2_O_2_ etching reaction in the presence of Cu^2+^. The longitudinal SPR band of the AuNRs shifted gradually to longer wavelengths as the concentration of CTAB increased from 1 to 40 mM. 

**Figure 9:**
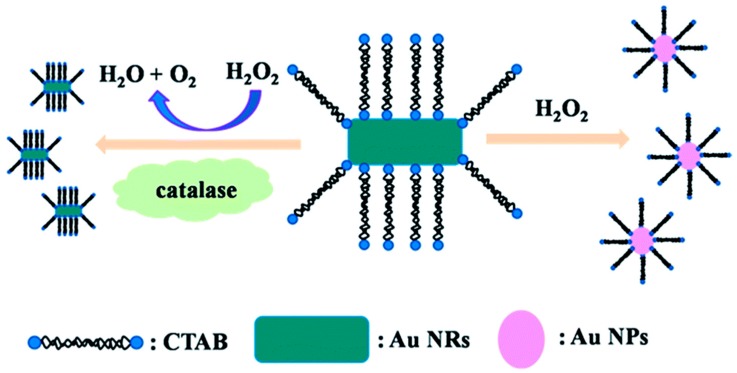
The schematic illustration for colorimetric detection of catalase based on the decomposition of H_2_O_2_, which inhibits H_2_O_2_ etching of AuNRs. Reproduced with permission from Reference [97]. Royal Society of Chemistry, 2016.

Except for using metal ions as a catalytic medium, enzymes could remarkably enhance the efficiency of the etching reaction with fast reaction kinetics. Saa et al. [95] reported that the H_2_O_2_-triggered etching of AuNRs caused a considerable change in the color from pink, blue to yellow, as a function of glucose concentration by using horseradish peroxidase (HRP). Consequently, this method had high sensitivity with a detection limit of 10 μM in a 15 min detection time. Saa et al. [96] also designed an ultrasensitive colorimetric sensor for the enzymatic activity of acetylcholinesterase (AChE) based on protecting the AuNRs against enzymatic etching by using thiol molecules. The absence of AChE made the shortening of AuNRs by the HRP-accelerated oxidative etching. However, with the addition of the AChE, acetylthiocholine (ATCh) in the solution was hydrolyzed by AChE to produce thiocholine. Thiocholine molecules then bound to the tips of the AuNRs, which led to a higher surface coverage of thiol molecules at the terminal end of the AuNRs. This reaction gradually decreased the rate of anisotropic oxidation of AuNRs by HRP as the AChE concentration increased, accompanied by a color change from blue to brown. The resulting LOD was 0.04 mU/mL which is lower than that of other currently reported AChE sensors. The authors also demonstrated the detection of AChE inhibitors such as paraoxon and galanthamine in nanomolar concentrations using this strategy. Lu et al. [97] developed the visual detection of catalase based on the inhibition of a H_2_O_2_-mediated etching reaction (Figure 9). In the absence of the catalase, the etching of AuNRs occurred and spherical AuNPs were obtained. Under these conditions, the solution had a pink color. In the presence of the enzyme, the decomposition of H_2_O_2_ to H_2_O and O_2_ was catalyzed, which slowed down the etching kinetics and resulted in the AuNR shape remaining nearly unchanged. This assay showed a better sensitivity to the detection of catalase than other sensors, and it was also used in a lab-based test that simulates real test conditions. 

### 3.2. Ion-Mediated Etching Reactions

**Figure 10:**
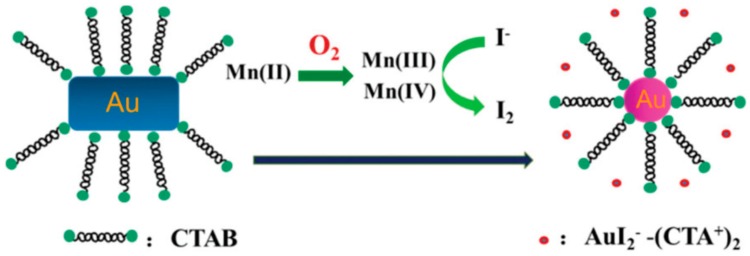
The schematic illustration for the detection of dissolved oxygen based on the iodine-mediated etching of AuNRs. Reproduced with permission from Reference [107]. Royal Society of Chemistry, 2016.

It has been reported that halide ions increase the oxidative etching of AuNRs efficiently because they promote the solubility of gold monoxide [98,99], particularly in iodine-mediated etching [100]. Iodine, acting as an oxidant, asymmetrically etches AuNRs in the presence of cetyltrimethylammonium (CTA^+^) ions due to the low surface passivation at the end terminals, which leads to an AuNR shape conversion and color change [101,102,103,104]. For example, the sensitive visual detection of molybdate was reported using the iodine-mediated etching assay [105]. Although H_2_O_2_ could oxidize I^−^ to I_2_ which caused the corrosion of CTAB stabilized AuNRs in the presence of a weak acid solution, the reaction kinetics was very slow. The addition of molybdate, which is a peroxidase-like molecule, resulted in the acceleration of the reaction between H_2_O_2_ and I^−^ to produce large amounts of I_2_ and the effective etching of AuNRs along the longitudinal direction. Good specificity and high sensitivity with a LOD of 1 nM molybdate were all achieved. This proposed strategy was considered advantageous compared to previous molybdate biosensors as it comprised a simple design, was cost-effective and did not require labeling. Further, on the basis of this phenomenon, Zhang et al. [106] reported a colorimetric glucose sensor based on the molybdate-promoted etching of AuNRs. In the presence of glucose, glucose oxidase (GOx) can catalyze the oxidation of glucose to generate gluconic acid and H_2_O_2_. In the presence of molybdate, H_2_O_2_ immediately oxidized I^−^ to I_2_, which etched the AuNRs in the longitudinal direction. In this case, the LSPR peak shift was directly dependent on the concentration of glucose in the sample. Under optimal conditions, this assay exhibited a good linear relationship in the range from 0.3 to 1 μM with a LOD of 0.1 μM. In another example, a colorimetric assay that relies on the principle of the I_2_-mediated etching strategy has been investigated for the on-site detection of dissolved oxygen [107]. In this study, Mn^2+^ was first oxidized to Mn^3+^ and Mn^4+^ and after acidification, I^−^ was oxidized to form I_2_ by Mn^3+^ and Mn^4+^ and an obvious color change from blue to red was observed (Figure 10).

In addition to halide ions, Cu^2+^ was found to promote significant AuNR etching by dissolved oxygen, which has a strong affinity for the Au surface atoms to form the stable Au–O complex [108]. The addition of Cu^2+^ helped etch the Au–O complex on the AuNR surface, and promoted electron transfer from Au to O. At Cu^2+^ concentrations lower than 1 mM, the end-cap reshaping conditions were satisfied, a slight etching reaction proceeded and the AuNRs quickly underwent a shape transformation. At higher concentrations of Cu^2+^, a variation in the end-cap shape distribution and an increase in the anisotropic etching of AuNRs were observed. Later, similar observations were also reported by Alex et al. [109], who studied the colorimetric detection of Cr^6+^ by Cr^6+^-assisted etching and reshaping of dumbbell-shaped AuNRs. The LOD of this strategy was 71 nM, which is lower than that of other colorimetric methods and comparable to the results obtained from fluorescence methods. Zhang et al. [110] developed a rapid, visual method to detect Cu^2+^ based on the catalytic etching of AuNRs. In the presence of CTAB, AuNRs were oxidized by Cu^2+^ to produce AuBr_2_–CTA^+^ and CuBr_2_-CTA^+^ complexes and thereafter the CuBr_2_-CTA^+^ complex as an ion-association agent was reduced by dissolved oxygen to produce Cu^2+^. Thus, there was no Cu^2+^ consumption in the etching process, which led to the maximal etching of the AuNRs. The system showed a significant improvement for practical applications in real samples like the sea and digested shellfish samples due to its good selectivity and high tolerance to the high salinity of these complex matrices. 

Additionally, Pd^2+^ have been reported to increase the etching rate of gold nanomaterials [111,112,113] but there are some drawbacks such as long reaction time, low sensitivity, and complicated process. Recently, Lan et al. [114] proposed a simple and fast Pd^2+^ sensor in complex real samples. They used S_2_O_3_^2−^ absorbed on the gold surface to induce a redox reaction at the solid–liquid interface due to the electrostatic interactions between S_2_O_3_^2−^ and the CTAB-capped AuNRs. This, in turn, induced the formation of an Au(S_2_O_3_)_2_^3−^ complex, which slowly dissolved the AuNRs. The etching rate was accelerated by adding Pd^2+^ because the quick formation of the Pb-Au alloy (AuPb_2_ and AuPb_3_) caused a significant reduction in the standard electron potential of gold in AuNRs and, therefore enhances the dissolution rate of the AuNRs. Under optimal conditions, this probe was highly sensitive (LOD = 4.3 nM) and selective toward Pb^2+^ ions within a rapid detection time (10 min).

## 4. Growth-Based Colorimetric Sensing Strategies

The growth of small-sized AuNPs on catalytic seeds such as Au^3+^ and Ag^+^ by enzymatic or chemical transformation is a new trend in designing a biological and chemical colorimetric sensing strategy. 

### 4.1. Non-Enzyme-Mediated Growth Reactions

**Figure 11:**
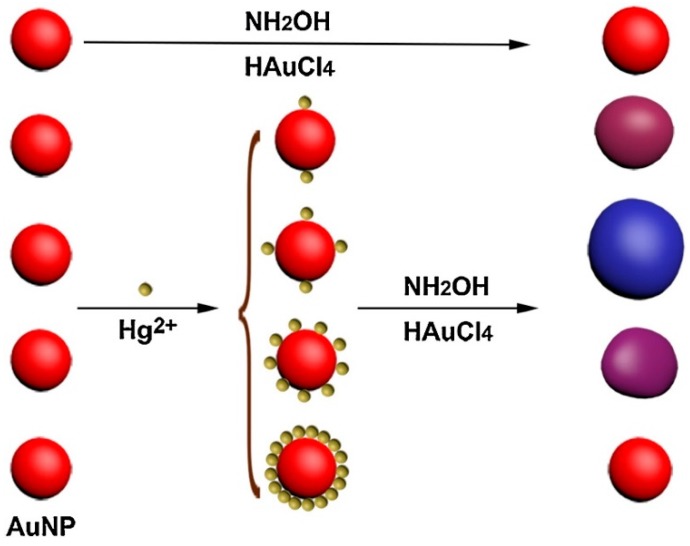
The schematic illustration of the amount of Hg on the surface of AuNP-controlled the growth of AuNPs. Reproduced with permission from Reference [118]. Elsevier B.V., 2017.

It is well-known that H_2_O_2_ can be used as the reductant for AuNP growth [115] or as the catalyst for the metallization of AuNPs [116]. Based on these principles, a colorimetric assay for metal ion detection was designed [117]. In the absence of the target Hg^2+^ ions, the decomposition of H_2_O_2_ was catalyzed by gold nanoclusters (AuNCs) and the Au crystal growth kinetics were slow, which led to the formation of crystals with an ill-defined morphology, including aggregated AuNPs. However, the addition of target Hg^2+^ ions inhibited the catalytic ability of the AuNCs toward H_2_O_2_ so the reduction of Au^3+^ with H_2_O_2_ occurs at a fast rate, and non-aggregated AuNPs were produced. Hydroxylamine (NH_2_OH) is another reductant which could thermodynamically reduce Au^3+^ to AuNP, and Hg^2+^ favorably absorbed on the Au surface to form a solid amalgam-like structure. Thus, Zhao et al. [118] combined these two phenomena for the development of a Hg^2+^ sensor. In this case, the growth of small-size AuNPs could be controlled by the amount of adsorbed Hg^2+^ on the AuNPs, and thus give rise to Au nanostructures of different sizes and solutions of different colors (Figure 11). The major feature of both studies is the direct colorimetric detection of Hg^2+^ without any ligand labeling. 

Researchers have also explored different sensing methods for proteins and small molecules in addition to metal ion detection. Shen et al. [119] developed a rapid colorimetric detection of tetracycline broad-spectrum antibiotics based on the direct reduction of Au^3+^ into AuNPs without added AuNP seeds. Tetracycline antibiotics enable the reduction of Au^3+^ to atomic Au, which could form AuNPs spontaneously, with the oxidation of the phenol group on the benzene ring. As the concentrations of tetracycline increased, the SPR peaks of the AuNPs were intensified and slightly blue-shifted. Although they provided a fast method for tetracycline antibiotics, this assay could not distinguish between different families of tetracycline. Li et al. [120] investigated the effect of AuNP particle sizes on their performance as colorimetric lysozyme probes. They found that the smaller size (15 nm) of AuNPs can be aggregated by the addition of lysozyme due to the Au–NH_2_ bonds but the sensitivity was unsatisfactory. Thus, a growth-based colorimetric method was performed to improve the sensing performance of this assay by using NH_2_OH as a reductant, which showed a good sensitivity with a LOD of 0.1 nM.

Recently, DNA molecules have been reported to affect the diffusion of Au^3+^ to the seed and control the morphology of AuNPs [121,122]. Some researchers have combined DNA aptamers with colorimetric diagnostics using the DNA-mediated growth system. Soh et al. [123] described a detection method for ochratoxin A (OTA) by using aptamer-controlled AuNP growth. The as-prepared AuNPs were capped with DNA aptamers through physical adsorption and the amount of aptamer strands were controlled by the specific aptamer–target interaction depending on the target concentration; the lower the OTA concentration, the higher the amount of capped aptamer. Hence, AuNPs with high aptamer coverage exhibited a branched morphology and blue solutions, whereas those with low aptamer coverage had a smooth, spherical morphology and red solutions. The result could be observed visibly by a change in the color of the solution from red to blue and quantified via absorbance spectroscopy. Likewise, Zhu et al. [124] employed this aptamer-mediated control of AuNPs morphology for the naked-eye detection of cholic acid.

### 4.2. Enzyme-Mediated Growth Reactions

It has been demonstrated that the reduction of Au^3+^ to AuNPs or the deposition of metals on AuNP seeds can be catalyzed by enzymes. For example, Liu et al. [125] reported a GOx-mediated nanocrystal growth for the attomolar detection of prostate-specific antigen (PSA). Here, secondary antibody (Ab2)–GOx conjugate functionalized magnetic beads were used as a capture probe, and primary antibodies (Ab1) were used as a detection probe for the specific recognition of PSA. Because solutions of small-sized AuNPs in low concentrations (<10 nM) are generally almost colorless, the change in size from 5 nm to larger sizes (>10 nm) can cause an obvious color change from colorless to red. Therefore, after the formation of immunocomplex (Ab1–PSA–Ab2-GOx), GOx triggered the oxidation of glucose to produce H_2_O_2_, which resulted in the enlargement of 5 nm AuNPs and the obvious color change from colorless to red. This assay showed a LOD of 93 aM, which is more than four orders of magnitude higher than that of the commercial ELISA method.

**Figure 12:**
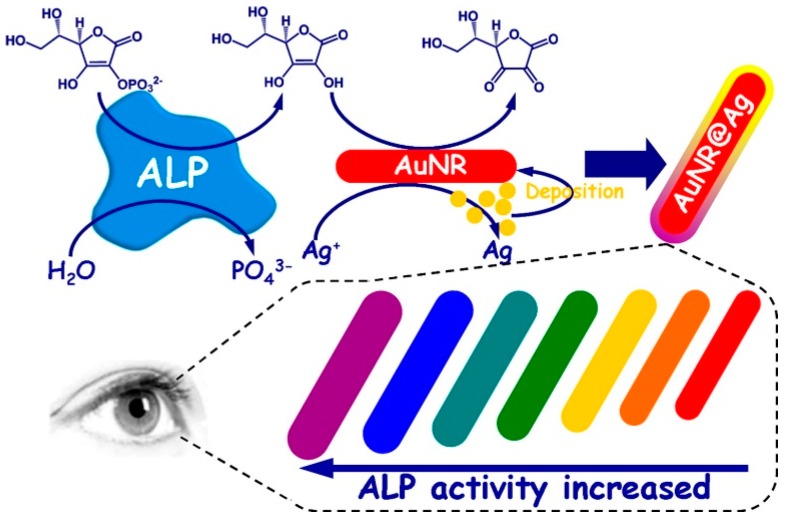
The schematic illustration of the colorimetric sensing of phosphatase activity based on enzymatic reaction-aided silver deposition onto AuNR to generate different color changes. Reproduced with permission from Reference [121]. American Chemical Society, 2014.

An enzymatic silver deposition assay based on the reaction between a certain enzyme, AuNPs and Ag^+^ to generate a color change has been extensively used for different applications [126,127,128]. Gao et al. [126] developed a high-resolution colorimetric assay for monitoring alkaline phosphatase (ALP) activity using enzyme-guided in situ formation of a silver shell on AuNRs (Figure 12). In this approach, the LSPR of AuNRs was tuned by the ALP-assisted crystal growth to favor the formation of a Ag^+^ coating around the AuNRs, which was accompanied with a perceptible color change from red, orange, yellow, green, cyan, blue and to violet with a high resolution. ALP produced ascorbic acid by hydrolyzing ascorbic acid 2-phosphate, which subsequently reduced the Ag^+^ to metallic silver on the surface of AuNR. The color change and the shift in the SPR peak were proportional to the size of the silver nanoshell, which indirectly depended on the ALP activity. They experimentally demonstrated the significant ability of the metallization of the AuNRs to detect the enzyme activity of ALP down to 14.5 mU/mL in serum. In other studies, similar concepts but using different shapes (sphere [129], rod [130], star [131], and bipyramid [132]) of gold nanomaterials have been reported for the detection of protein targets.

## 5. Nanoenzyme-Based Colorimetric Sensing Strategies

Recently, AuNPs with different surface modifications have been found to possess an intrinsic enzyme-mimetic activity, which provides new insights into the catalytic application of this nanomaterial in colorimetric biosensing. Compared with natural enzymes, artificial AuNP enzymes have several advantages, such as high stability against denaturing, easy synthesis, and facile storage. Therefore, applications of AuNPs as artificial enzymes are currently under investigation.

### 5.1. Peroxidase-Like Activity

Positively- and negatively-charged AuNPs have been reported to possess intrinsic peroxidase activity and have been used in biochemical analyses [133,134]. The charged AuNPs can catalyze the oxidation of substrate 3,3,5,5-tetramethylbenzidine (TMB) in the presence of H_2_O_2_ to generate a blue color in aqueous solutions. Thus, monitoring the color change of TMB could provide an indirect method to fabricate colorimetric assays for substrates. For example, Jiang et al. prepared chitosan-functionalized (positively-charged) AuNPs which exhibited better peroxidase-like activity than that exhibited by natural enzymes [135]. Additionally, glucose was detected in a buffer system with a LOD of 3 mM and in 60% serum with a LOD of 12 mM. In a further study, they found that Hg^2+^ could significantly enhance the catalytic ability of chitosan-functionalized AuNPs [136], and constructed an assay for Hg^2+^ detection. The Hg^2+^ detection method had a linear range of 0.04–10.2 nM with a detection limit of 20 nM. Wu et al. synthesized 2,6-diaminopurine (DAP)-capped AuNPs which exhibited peroxidase-like activity [137]. The activity of DAP–AuNPs can be improved by Fe^2+^. The hydroxyl radicals might be produced effectively by the complex of DAP–AuNPs and Fe^2+^, which led to the enhancement of the catalytic activity. Therefore, a sensitive Fe^2+^ sensor was reported with a LOD as low as 1.28 nM. Because hemoglobin (HB) with O_2_ binds to Fe^2+^ in red blood cells (RBC), this assay was applied for the detection of HB and RBCs in urine samples.

**Figure 13:**
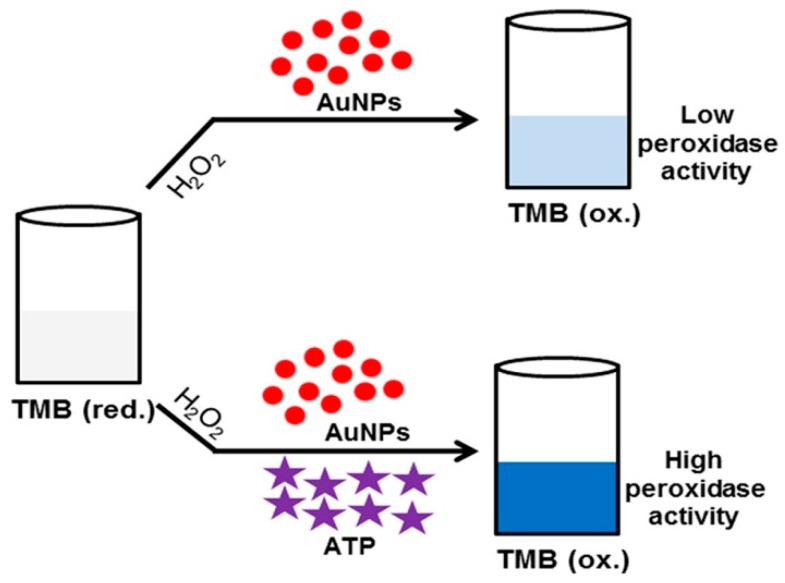
The schematic illustration of the colorimetric sensing mechanism for adenosine triphosphate (ATP) based on the ATP-promoted nanozyme activity of AuNPs. Reproduced with permission from Reference [138]. Elsevier B.V., 2015.

Notably, the peroxidase-like catalytic activity is affected by the surface properties of the AuNPs and the particle-mediated electron transfer processes. The positively-charged AuNPs have superior peroxidase-like activities compared to the negatively-charged AuNPs. However, several molecules can be used for promoting activities of negatively-charged AuNPs. Therefore, on the basis of their effects on the activities of negatively-charged AuNPs, several targets have been detected. Shah et al. reported that adenosine triphosphate (ATP) could significantly improve the peroxidase-like activity of citrate-capped AuNPs [138], and developed an assay for detecting ATP (Figure 13). By contrast, many groups found that the absorption of DNA aptamers on AuNPs inhibited the activities of citrate-capped AuNPs [139,140]. Like a competitive inhibition approach, the presence of the target could allow the aptamer to detach from the AuNP surfaces in a target concentration-dependent manner, which resulted in the reactivation of the peroxidase-like activity of the AuNPs. Likewise, the catalytic activity could be modified by the amount of cysteine [141]. However, the optimal catalytic activity of AuNPs was in acidic conditions (around pH 4), which restricts their practical applications. 

### 5.2. Other Enzyme-Like Activity

**Figure 14:**
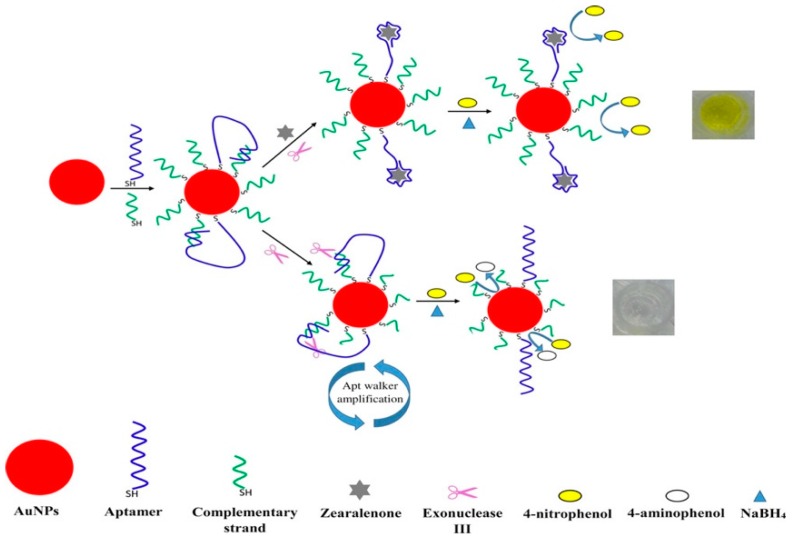
The representation of the detection of zearalenone based on the exonuclease III-aided aptamer walker and catalytic reaction of AuNPs. Reproduced with permission from Reference [147]. American Chemical Society, 2018.

In addition to the peroxidase-like activity, AuNPs have also been demonstrated to have other enzyme-mimicking activities for colorimetric assay applications such as glucose oxidase, superoxidase dismutase, and catalase-like activity. Zhao et al. synthesized supramolecular functionalized AuNPs which possessed two enzyme-like properties using cyclodextrin as both the stabilizer and the reducing agent [142]. Moreover, the cyclodextrin-modified AuNPs were rather robust for the cascade reaction; reactions from glucose to gluconic acid and H_2_O_2_ (glucose oxidase-mimicking activity), to H_2_O and oxidized TMB (peroxidase-mimicking behavior), were catalyzed by the sole supramolecular functionalized AuNPs. Chen et al. developed a simple colorimetric strategy for protein targets using aptamer–gold nanoparticle conjugates [143]. These conjugates included catalytically active AuNPs for the amplification of the colorimetric readout response and anchored aptamers for target recognition. In the presence of thrombin, the aptamer–thrombin interaction effectively masked the catalytic AuNP surface and hampered the access of 4-nitrophenol to the surfaces of AuNPs. Without thrombin, however, yellow 4-nitrophenol could freely attach to the catalytic surface and turn to colorless 4-aminophenol. Based on this mechanism, the LOD was 0.1 nM with the naked eye, which indicated an ultrahigh sensitivity assay. Similar approaches based on the conversion of 4-nitrophenol to 4-aminophenol had been reported for the detection of other protein targets [144,145,146]. Last year, Taghdisi et al. developed the amplified colorimetric aptasensor for zearalenone based on the exonuclease-aided aptamer walker and the catalytic AuNPs [147]. Without targets, the aptamer bound to complementary strands of aptamer on the AuNP surface and complementary strands would be degraded by the introduction of exonuclease, which led to the unmasking of the AuNP surface. As a result, 4-nitrophenol could approach the AuNP surface easily and induce a color change from yellow to colorless (Figure 14). The strategy for the detection of zearalenone in a serum sample has also been proved. 

## 6. Summary and Outlook

The development of a simple and convenient biosensor for the detection of chemical and biological agents is of significant importance. Compared with other sensing methods, AuNP-based colorimetric assays are promising because the whole assay proceeds through a simple solution of the target and probes without the washing steps, and the color change can be directly monitored with the naked eye without sophisticated instruments. Therefore, these approaches remarkably simplify the operation procedures, shorten the detection times, and significantly reduce the assay costs. Moreover, such colorimetric assays are easily adaptable to smartphone-based devices, which is a potentially powerful platform to detect, transduce and analyze on-line sensing information [148,149,150]. Obviously, it will be a good way to push colorimetric sensors forward with the integration of smartphone-based technique. In this review, we reported the recent developments in AuNP-based colorimetric methods including aggregation, etching, growth, and nanozyme for sensing applications. A large number of selected examples of AuNP colorimetric assays have been introduced, and some novel and interesting strategies with an outstanding sensor performance have been discussed. However, there are still some critical issues that need to be addressed in the future. First, for many analytes such as tumor markers or antibiotics, the concentrations are commonly below the critical threshold levels, at which point the biological and chemical targets are often undetectable by using current AuNP-based platforms. To improve the sensitivity, various signal amplification methods have been rationally designed and combined with the AuNP-based colorimetric approaches. However, these amplification processes usually prolong the detection time and increase the experimental complexity. Thus, one of the future directions of the field is to develop ultrasensitive AuNP-based colorimetric sensors without using amplification processes. Second, the simultaneous detection of multiple analytes in the same solution has attracted much attention because this method provides a significant advantage for rapid, simple, and reagent consumption. Although AuNP-based colorimetric sensors are extremely useful and simple, they are usually only employed in single-target assays. Advances in material science and analytical techniques might design a new multicolor AuNP sensor to detect multiple targets in a one-pot reaction. Last but not least, although large-scale production of AuNPs for colorimetric sensing applications is well-established, their practical use in industry has not been explored yet. One possible reason is that many AuNP-based colorimetric assays are still at the proof-of-concept stage, and there is a notable difference in the sensitivity and specificity of assays for analytes in pure systems versus in complex samples. Furthermore, the background color of real sample solutions such as human serum or industrial wastewater may interfere with the colored signal, which affects the accuracy of the assay. Therefore, further explorations are required to investigate the possible applications of AuNP-based colorimetric assays in clinical medicine and industry. 

Typically, the development of AuNP-based colorimetric nanosensors has continued at a fast pace. Even though challenges still exist, there is a scope for future research and the practical applications of colorimetric strategies in environmental monitoring, chemical detection, biomedical analysis, and healthcare diagnoses still need to be developed.
